# Microwave-assisted acid treatment for the mineral transformation of chrysotile as an alternative for asbestos waste management

**DOI:** 10.1007/s10653-024-01993-6

**Published:** 2024-07-18

**Authors:** Salima Essih, Laura Pardo, Juan Antonio Cecilia, Lucía dos Santos-Gómez, Rosario M. P. Colodrero, Manuel Pozo, Gustavo Calero, Francisco Franco

**Affiliations:** 1 Present Address: Departamento de Química Inorgánica, Cristalografía y Mineralogía, Facultad de Ciencias, Campus de Teatinos, Universidad de Málaga, 29071 Málaga, Spain; 2 Departamento de Geología y Geoquímica, Facultad de Ciencias, Universidad Autónoma de Madrid, Cantoblanco, 28049 Madrid, Spain; 3 Aguas de Torremolinos, CL. Periodista Federico Alba, 7, 29620 Torremolinos, Málaga Spain

**Keywords:** Acid treatment, Microwave, Asbestos, Chrysotile, Waste management

## Abstract

In this work, the effect of microwave-assisted acid treatments on the morphological and crystallochemical characteristics of chrysotile fibers is investigated. A low concentration of nitric acid (0.2 N) is used to remove Mg^2+^-species located in the octahedral sheet of its structure, thereby causing a crystallo-chemical change forming a skeleton of non-crystalline amorphous silica. This skeleton maintains an elongated morphology but characterized by rounded -not sharp-edges and porous surfaces whose physical resistance under stress is reduced when compared with the initial fibers of chrysotile, favoring a lower pathogenicity of the fibers. Thus, microwave-assisted acid treatment rise as a low-cost, fast and effective option in avoiding the dangerousness associated with asbestos waste management.

**Supplementary Information:**

The online version contains supplementary material available at 10.1007/s10653-024-01993-6.

## Introduction

The World Health Organization estimates that 125 million people around the world are exposed to inhalation of asbestos fibers in the workplace, and more than 107 000 people die from asbestos-related diseases, including lung cancer, mesothelioma or asbestosis, due to asbestos exposure (Douglas & Van den Borre, 2019; Landrigan et al., 2019; Lemen, 2004; Park, 2018; Suzuki et al., 2005; Yarborough, 2007). Asbestos has been used for more than 2500 years and has had a very diverse use due to the advantages related to its chemical inertness, its properties as a thermal insulator and its flexibility (Cortés, 2003; Langer et al., 1991). Other applications of asbestos in the industry have been its use in ventilation systems, construction, railways, textiles and friction materials such as vehicle parts, brake pads and clutch discs (Fernández et al., 2013).

From a mineralogic point view, asbestos are minerals belonging to the class of silicate with a fibrous habit derived from their crystallo-chemical characteristics (Mazzeo, 2018). The minerals of the asbestos group are classified into two types of silicates: inosilicates and phyllosilicates (Community Health, 1983). Within the inosilicates, the amphibole group presents minerals that are considered asbestos, i.e. amosite (brown asbestos, very long but very hard fibers), crocidolite (blue asbestos, intermediate hardness between chrysotile and amosite), tremolite (another variety of white asbestos), actinolite (white color, little used) and anthophyllite (white color, masses of very short fibers) (Community Health, 1983). Among fibrous phyllosilicates only chrysotile (serpentine group) is well known as white asbestos with ideal formula Mg_3_Si_2_O_5_(OH)_4_ (Thives et al., 2022). Chrysotile is the most flexible asbestos and is therefore the most used for spinning and weaving (Rudd, 2008).

Chrysotile is a 1:1 phyllosilicate. with a layered structure (Langer et al., 1991). The sheets are made up of magnesium and silicon atoms that are bonded together in a hexagonal lattice. The sheets are held together by weak bonds, which makes them easily separated. This is why chrysotile can be easily crumbled into fibers. Its curved laminar structure generates particles with an achiral fibrous morphology normally rolled in the a-axis or, less commonly, in the b-axis (Brown & Brindley, 1980, Anbalagan et al., 2010). A tetrahedral [SiO_4_]^−4^ silicate sheet and a trioctahedral [Mg_3_O_2_(OH)_4_]^−2^ brucite-like sheet can be distinguished in this magnesian silicate (Anbalagan et al., 2010). According to the Stanton’s hypothesis, relatively long asbestos fibers (0.25 μm of section and 8 μm of length) are strongly carcinogenic, while fibers with shorter length showed less risk (Harington et al., 1971, 1975). However, Suzuki et al. (2005) performed a pathological analysis of human lung tissue with mesothelioma in 168 individuals to check Stanton's hypothesis. In this study, 10,575 asbestos fibers were identified in the tissues and only 2.3% of them had the dimensions of Stanton's hypothesis. Moreover, most of the fibers were less or equal to 5 μm, with a section less than 0.25 μm, and among the asbestos found in the tissue, chrysotile was the most common. Contrary to Stanton's hypothesis, these results suggested that short and thin asbestos fibers seem to contribute more to the formation of human malignant mesothelioma and should be considered as an important factor in the origin of asbestosis (Suzuki et al., 2005).

On the other hand, the storage of the asbestos without inactivation can generate future health problems (Peña-Castro et al., 2023). For this reason, it is necessary to carry out procedures allowing the inactivation of its dangerousness, so that the final waste can be safely stored (Spasiano & Pirozzi, 2017). Several processes for the inactivation of asbestos are currently known, i.e. mechanical (Bloise et al., 2018; Paolini et al., 2019), biological (Paolini et al., 2019), thermal (Bloise et al., 2016, 2018; Bloise 2023a,b ; Dellisanti et al., 2009; Gualtieri & Boccaletti, 2011a, 2011b, 2012; Kusiorowski et al., 2012, 2013; Paolini et al., 2019; Zaghloul & Circeo, 1993) and chemical treatments (Valouma et al., 2017). Among them, thermal treatment such as vitrification, ceramization, reminarization and steel casting, are the most employed procedures. These procedures are tedious and asbestos need to be heated at elevated temperature (1000–1600 ºC) to turn them into a more stable compound without fibrous morphology (Dellisanti et al., 2009; Paolini et al., 2019; Zaghloul & Circeo, 1993).

Another alternative to degrade the fibrous structure of aluminosilicates, which could be applied to deactivate the harmful effect of asbestos, is acid treatment (Komadel, 2016). Acid treatments on clays have been well known since the 1950s when dioctahedral smectites were treated with HCl causing a partial or total dissolution of the octahedral layer (Osthaus, 1953, 1955). Generally, these treatments are carried out with concentrated inorganic acids during long treatment periods (12–24 h) at temperatures between 60–90 ºC (Komadel, 2016). Regarding to chrysotile, several authors have pointed out that the exposition of chrysotile asbestos to organic and/or inorganic acids, with a pH < 6, the magnesium species of the chrysotile is leached due to the interaction between the hydrogen ions liberated from the acids and the hydroxyl groups in the Mg(OH)_2_ sheet (Cozak et al., 1983; Goni et al., 1979; Jaurand et al., 1981), leading to the inoculation of chrysotile. In the same way, Hongo (2016) has pointed out that the treatment below pH 2 the efficient of the chrysotile treatment damages the outer surface of chrysolite fibers more efficiently. Thus, previous studies have pointed out that the fluorosulfonic acid has enough potential to dissolve both the tetrahedral and octahedral sheet (Sugama et al., 1998). Other authors have carried out a comparative study to leach chrysotile in nitric, sulfuric and oxalic acid at pH = 1 although it is required about 9 days for the amorphization of chrysotile (Rozalen & Huertas, 2013). In the same way, other authors have reported that it is required concentrated solutions of H_2_SO_4_, HNO_3_ or H_3_PO_4_ to destroy de fibrous habits of chrysotile (Hongo, 2016; Nam et al., 2014; Pawelczyk et al., 2017). In most cases, the acid treatments are very severe causing a total leaching of the chrysotile instead of its amorphization.

More recently, it has been demonstrated that microwave-based heating methods can be successfully employed in the inactivation process of clay minerals, increasing the heating rate and, thus, reducing the treatment time and favoring a more homogeneous heating than conventional methods (Korichi et al., 2009). For instance, Korichi et al. (2009), Franco et al., (2014, 2016, 2020) and Pardo-Canales et al. (2020) reported that the amorphization time of clay minerals of smectite group can be reduced from 48 h to few minutes when microwave assisted acid treatment is employed. These authors pointed out a leaching of phyllosilicates when Mg-species are located in the octahedral sheet, i.e. for the octahedral sheets using very diluted acid concentrations (< 0.2 M) (Franco et al., 2014, 2016, 2020; Pardo-Canales et al., 2020). Some investigations have also confirmed that microwave treatment is a promising method for detoxifying chrysotile (Granat et al., 2015; Yoshikawa et al., 2015). However, they noted that more research is needed to fully understand the mechanism of action and to optimize the treatment conditions.

Considering these premises, in this work a new procedure based on the microwave-assisted acid treatment is developed to modify the composition, morphology and mechanical properties of another Mg-rich phyllosilicate, such as chrysotile, using shorter time of inactivation treatment in comparison to the traditional acid treatment (Hongo, 2016; Nam et al., 2014; Pawelczyk et al., 2017). Under these procedure, Mg-species located in the octahedral sheet must be leached, causing an increase of the surface area and pore volume, as was observed in other phyllosilicates (Franco et al., 2014, 2016, 2020). These modifications of the textural properties must cause changes in the fibrous habits of the chrysotile, minimizing its dangerousness. This new strategy opens new perspectives for the asbestos waste management. In the present study, a comprehensive analysis of the treated chrysotile sample is also carried out.

## Experimental

### Microwave-assisted acid treatment

Acid treatments were performed by immersing 0.13 g of chrysotile fibers in 50 mL of a HNO_3_ solution 0.2 N (Supplementary Material, Figure S1). A low concentration of the acid solution was chosen in order to avoid several neutralization steps of the liquid waste. This suspension was kept under microwave irradiation at 700 W for 8, 12, 16 and 20 min (Monowave 300, Anton Paar). During irradiation, samples were stirred at 1200 rpm to assure a good temperature homogeneity. After the microwave-assisted acid treatment, the solid was recovered by centrifugation at 8000 rpm for 4 min.

### Characterization of the samples

The natural chrysotile sample (Quebec, Canada) and those obtained after the microwave treatments were completely characterized. The structure of the samples was determined by X-ray diffraction (XRD), collected on a X'Pert PRO MPD automated diffractometer (PANalytical B.V.) equipped with a Ge (111) primary monochromator (CuK_α1_ radiation) and an X'Celerator detector. The overall measurement time was 33 min per pattern in order to obtain good statistic over the 2θ range of 2–65° with 0.017° step size. GSAS and X'Pert HighScore Plus software package were employed for the XRD data analysis (Amelo, 2012; Larson & Dreele, 1994).

Diffuse Reflectance Infrared Fourier Transform spectroscopy (DRIFT) spectra were collected on a Harrick HVC-DRP cell fitted to a Varian 3100 FT-IR spectrophotometer. The interferograms consisted of 200 scans and the spectra were collected using a KBr spectrum as a background.

N_2_ adsorption/desorption isotherms of the samples were measured at − 196 °C, using a Micromeritic ASAP 2020 apparatus. The samples were degassed at 200 ºC to desorb all the water molecules. The specific surface areas (S_BET_) were determined through the Brunauer–Emmett–Teller equation (Brunauer et al., 1938; Dogan et al., 2006), and the surface area of the micropores and the external surface area were calculated using the t-plot method.

The morphology of the starting chrysotile and the obtained materials were examined by scanning electron microscope (SEM) using a JEOL SM-6490 LV combined with X-ray energy dispersive spectroscopy (EDX). The samples were previously gold sputtered (10 nm thick) in order to achieve good conductivity and avoid charging of the surface.

## Results and discussion

### Structural characterization and textural properties of chrysotile fibers

#### XRD

**Fig. 1:**
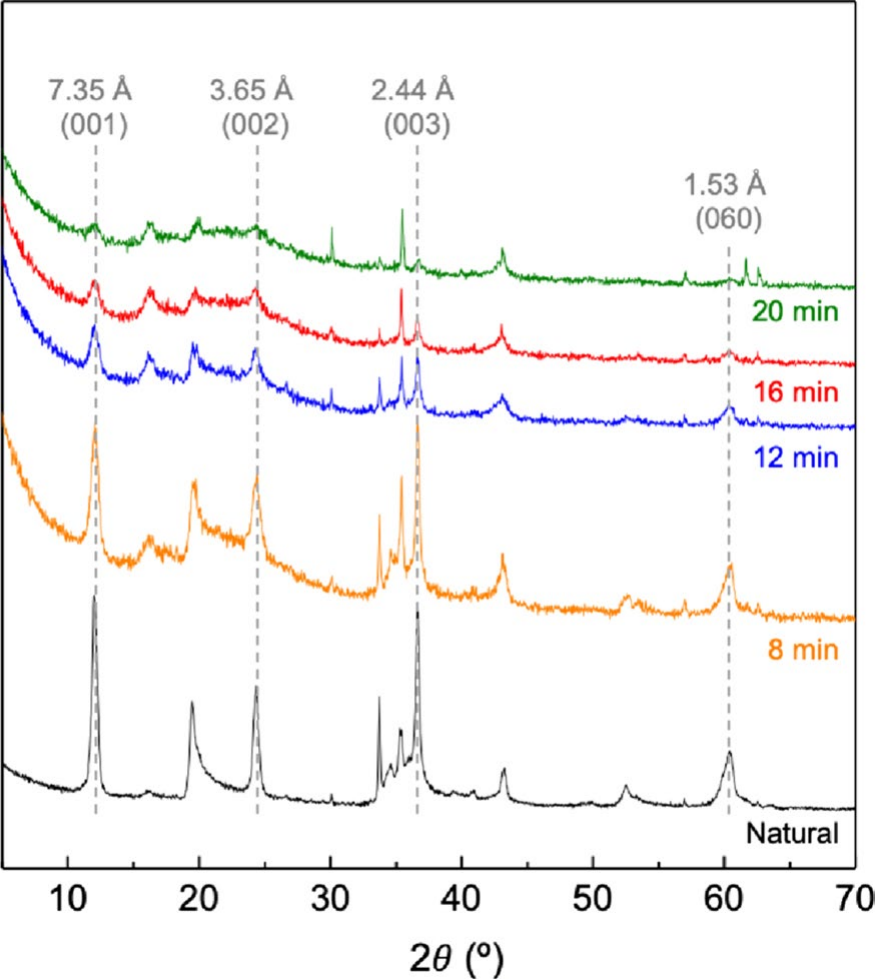
XRD patterns of natural and treated chrysotile after 8, 12, 16 and 20 min of microwave-assisted acid treatment

Figure 1 shows X-ray diffraction patterns of the natural chrysotile and the materials obtained after microwave-assisted acid treatments. The X-ray diffraction pattern of the natural chrysotile is dominated by the most intense peaks at 7.30, 3.65, 2.44 Å corresponding to the diffraction of the basal planes (00* l*) and a peak at 1.53 Å corresponding to the reflection d_060_, which shows that chrysotile is a trioctahedral phyllosilicate. This result is consistent with previous studies and shows that the basal space of chrysotile is slightly higher than those of other phyllosilicates with similar 1:1 layer structure, such as kaolinite whose basal space is 7.14 Å (Ruíz Cruz & Franco, 2000).

After 8 min of microwave-assisted acid treatment, the intensity of the peaks located at 7.35 and 3.65 Å decreases dramatically. These results indicate that the acid treatment has caused a strong amorphization of the structure that becomes more intense with the time of treatment, reaching the complete amorphization after 20 min. Moreover, a gentle increase of the background with the time of acid treatment is detected, confirming the presence of a non-crystalline phase, related with the presence of amorphous silica (Maletaskic, et al., 2018; Naumova, et al., 2019; Wang, et al., 2006).

#### DRIFT

**Fig. 2:**
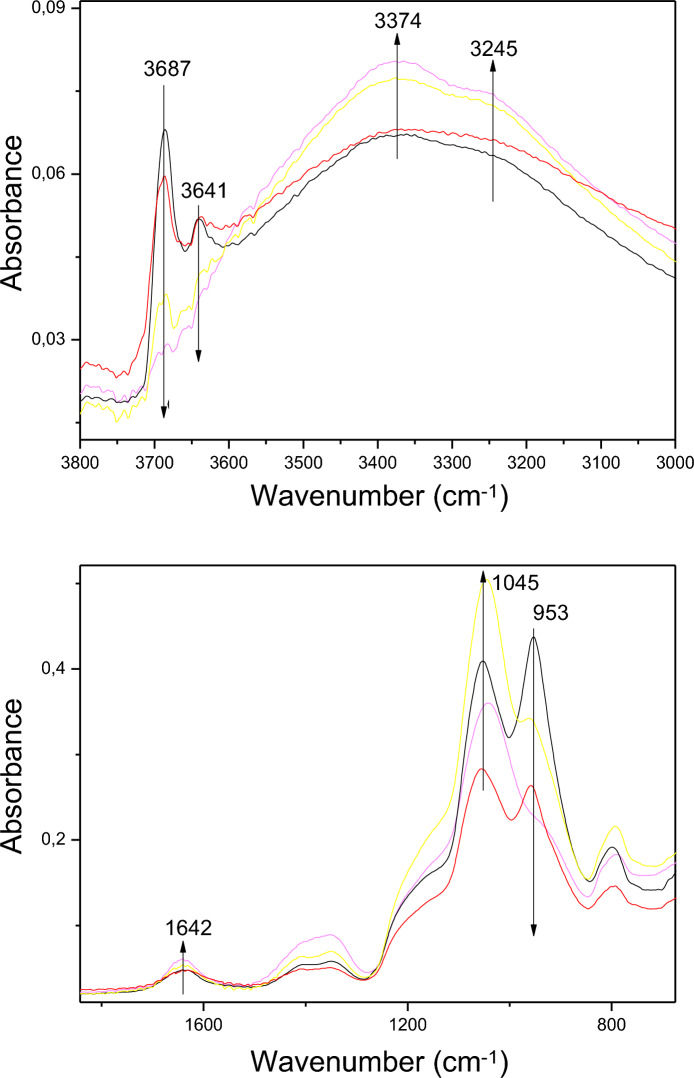
Infrared spectra of natural chrysotile (black) and chrysotile obtained after 8 (red), 12 (pink) and 20 min (yellow) of microwave-assisted acid treatments

The regions between 3800–3000 and 1800–800 cm^−1^ of the DRIFT spectra of the natural and treated chrysotile obtained after microwave-assisted acid treatments are shown in Fig. 2. Natural chrysotile present two bands at 3687 and 3641 cm^−1^ corresponding to the stretching vibration of the hydroxyl groups coordinated with magnesium atoms (Anbalagan et al., 2010; Franco et al., 2006). Moreover, wide bands centered at 3374 and 3245 cm^−1^ are also observed, which have been classically ascribed to the stretching mode of water molecules, which normally appears adsorbed at the external surface of particles.

After the acid treatments, there is a decrease in the bands corresponding to the OH groups and an increase in the intensity of the OH^−^ stretching band of adsorbed water molecules. After 20 min of treatment, the bands at 3687 and 3641 cm^−1^ have practically disappeared. This shows that after this time, the octahedral sheets in the chrysotile structure have practically disappeared. The bands corresponding to the stretching vibrations of the Si–O bonds appear at 953 and 1045 cm^−1^ (Franco et al., 2006). The band at 953 cm^−1^ decreases drastically after the treatments, while the band at 1045 cm^−1^ increase due to the Si–O band of amorphous silica. These results confirm that the chrysotile structure is strongly affected by microwave-assisted acid treatments and a new amorphous silica phase is generated. In addition, there is an increase in the 1642 cm^−1^ band, which is related to the deformation vibrations of the water molecules adsorbed at the surface of the amorphous silica.

#### Textural properties

Textural parameters of natural and treated chrysotile were determined by N_2_ adsorption/desorption isotherms at -196 ºC (Supplementary Information, Figure S2). In all cases, isotherms can be classified as type II, which are typical of either non-porous or macroporous materials according to the IUPAC classification (Sing et al., 1985). Regarding to the hysteresis loop, the N_2_ adsorption/desorption isotherms can be considered as type H4, which is usually found in solids with narrow pore diameter as some zeolites or micro-mesoporous carbons (Thommes et al., 2015). The loops are more pronounced in the case of the sample undergone to the microwave-assisted acid treatment, which suggests the formation of micro and/or mesopores by a partial leaching of the chrysotile.

The specific surface area data indicate that the starting chrysotile has hardly any porosity, as indicated the small amount of N_2_ adsorbed at low relative pressures (Fig. 4). On the other hand, an increase in the amount of N_2_ adsorbed at higher relative pressure can be observed, which is attributed to the N_2_ adsorbed between adjacent particles.

The specific surface area (S_BET_), the surface area of the micropores (S_micro_) and the external surface area (S_ext_) values for the untreated and treated chrysotile are shown is Table 1. After the microwave-assisted acid treatments, a clear increase in the S_BET_ is observed, ranging from 10.0 to 248.5 m^2^ g^−1^ for the untreated and 20 min-treated samples, respectively, due to the fact that the partial dissolution of chrysotile generates mesopores and mainly micropores. This is also confirmed by the S_micro_, which reaches its highest value of 138.2 m^2^ g^−1^ for the chrysotile treated for 20 min. The S_ext_ increases with the time of the acid treatment, and presents lower values than S_BET_ for treated samples, suggesting that all specific surface area is not equally accessible. Moreover, S_BET_, S_micro_ and S_ext_ for chrysotile treated for 16 and 20 min are very similar, showing that the surfaces of the samples are practically unaffected for acid treatments higher than 16 min. These data are similar to those reported by previous authors where mesoporous silica fibers were also obtained by acid treatment of chrysotile leaching although it is required at least 4 h of treatment and more concentrated acid solutions (Maletaskic, et al., 2018; Wang, et al., 2006).

**Table 1:** Specific surface area, surface area of the micropores and external surface area for the natural and treated chrysotile for 8, 12, 16 and 20 min

	Natural	8 min	12 min	16 min	20 min
S_BET_ (m^2^ g^−1^)	10.0	146.1	160.8	249.2	248.5
S_micro_ (m^2^ g^−1^)	0.0	63.5	79.3	130.6	138.2
S_ext_ (m^2^ g^−1^)	10.0	82.6	81.5	118.5	110.3

### Morphological characteristics of chrysotile fibers

**Fig. 3:**
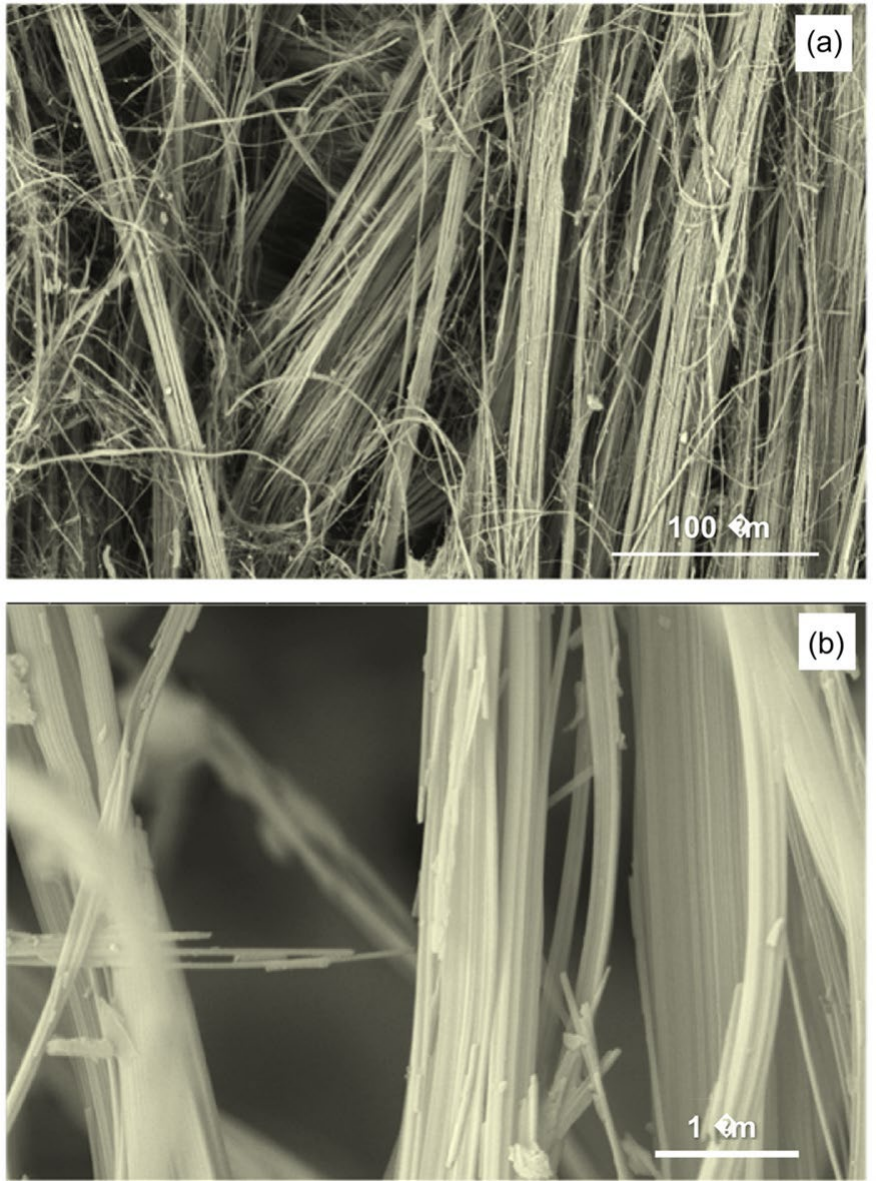
SEM images of natural chrysotile fibers at different magnifications

Figure 3 shows SEM images of natural chrysotile fibers, which have a strong anisotropic characteristic since the thickness of the fibers (< 0.001 mm) is extremely small compared to their length. These individual fibers are associated forming parallel bundles with sections between 0.01 and 0.05 mm. Moreover, the fiber density in the aggregates is not homogeneous, presenting zones with a high fiber density but very low density in others. Chrysotile has a fibrous habit with extremely fine fibers forming bundle of at least 50 units with thickness varying from 1 to 50 µm. Furthermore, some fibers can have a certain curvature due to bundles of fibers are progressively frayed by the acid treatment. It can be seen how the fibers have an extremely smooth surface texture with little porosity on the surface.

**Fig. 4:**
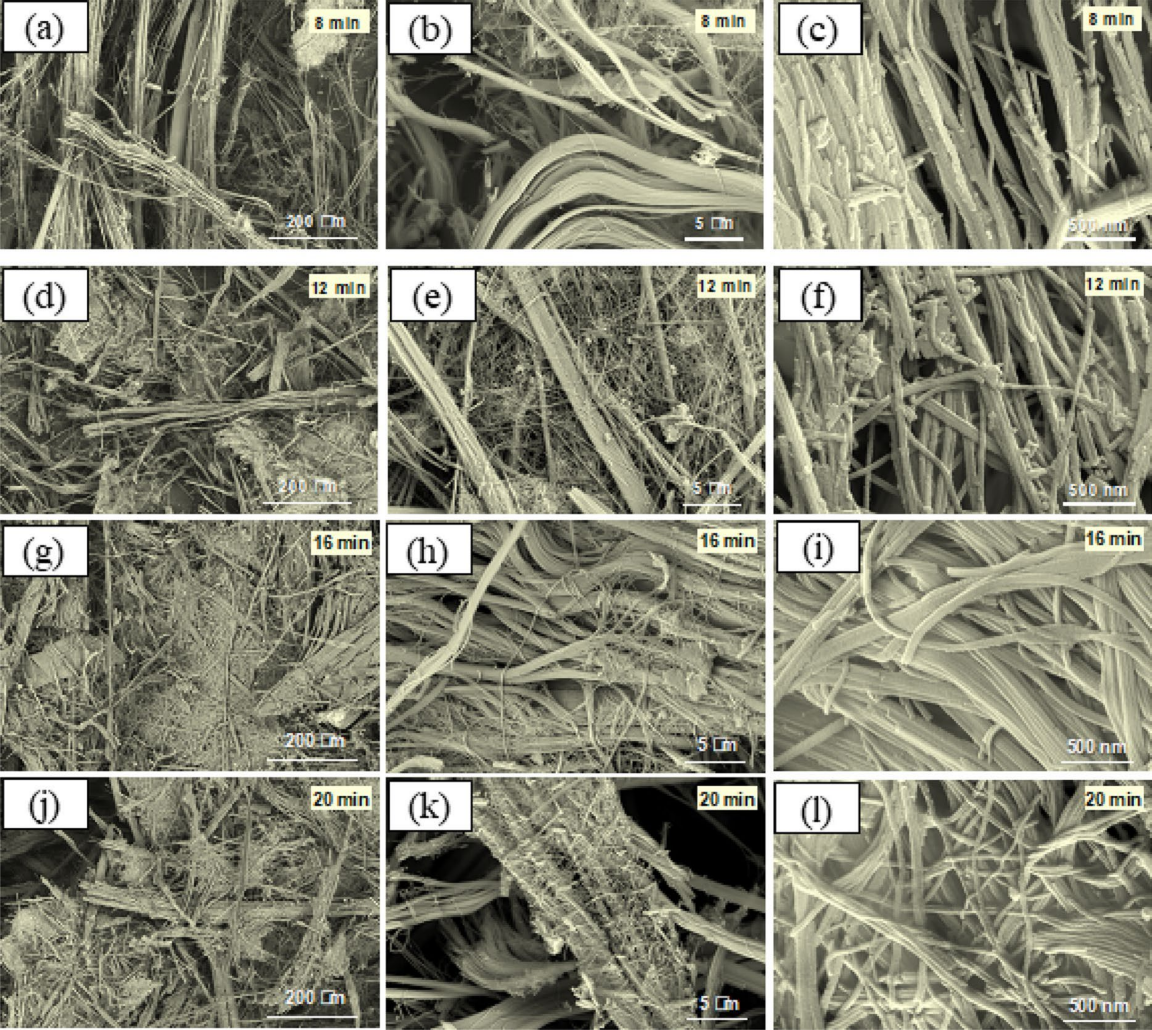
SEM images of treated chrysotile fibers for **a**–**c** 8 min and **d**–**f** 12 min, **g**–**i** 16 min and **j**–**l** 20 min

Figure 4 shows SEM images of the chrysotile fibers treated with a microwave-assisted acid approach at 8 and 12 min. In these cases, a strong exfoliation is clearly observed in a certain number of skeins. After 8 min of treatment (Fig. 4a–c), the number of isolated fibers is greater than in the case of natural chrysotile, where they all seem to be grouped in parallel forming skeins. These fibers have already lost their fibrous habit, decreasing their anisotropy compared to the natural sample without treatment. In addition, it can be distinguished the accumulation of particles smaller than 0.1 µm adhered to the surface of the fibers, unlike the untreated natural ones. This may simply be due to the fact that the microwave-assisted acid treatments produce a small fragmentation of the fibers, which generates a small residue that is deposited on the surface of the long fibers.

The morphology of the chrysotile fibers treated for 12 min has changed completely (Fig. 4d–f). Fibers that initially appeared straight have completely disappeared, suggesting that these fibers are more fragile and can be fragmentated more easily. In addition, these fibers appear absolutely dispersed with lengths between 0.5 and 3 µm and thickness around 0.2 µm. The strong anisotropy that chrysotile fibers initially present has been reduced with these treatments. Furthermore, most of them present an aspect that ceases to be fibrous, appearing as small particles grouped together with a slightly more spherical morphology.

SEM images of chrysotile fibers after 16 and 20 min microwave-assisted acid treatment are shown in Fig. 4g–l. The surfaces of such samples are very rough, showing a great porosity and the edges are very rounded. In addition, a large number of these fibers are no longer straight, a characteristic of chrysotile fibers before treatment, and seem to present a greater weakness due to the elevated number of isolated particles.

The chemical composition of natural chrysotile samples and those modified by microwave-assisted acid treatments were analyzed by EDX. Twenty-five-point analyses were performed on each sample to determine the average values of the chemical composition of all the materials analyzed (Table 2). The ratio of octahedral cations (Mg + Al)/Si is 1.46 for natural chrysotile. It can also be observed certain percentages of aluminum and calcium, due to the presence of other minor minerals as calcite or feldspars. Furthermore, the chemical composition of the treated samples strongly changes with only 8 min of treatment since the ratio between the octahedral cations and silicon decreases up to 0.51. This ratio continues to diminish as the acid treatment time increases, reaching a minimum of 0.12 after 20 min of treatment. Thus, magnesium, aluminum and calcium are dissolved with acid treatments due to the fast dissolution of the calcite detected as impurity and a more progressive leaching of the Mg-species located in the octahedral sheet of chrysotile as well as Al-species detected in feldspars. However, the silica content remains constant, leaving an elongated skeleton of silica with greater rigidity compared to the natural chrysotile fiber. The fast dissolution of the Mg-species in the octahedral sheet agrees with other data reported in the literature using a traditional acid treatment although these studies required longer reaction times and more concentrated acid solutions (Maletaskic, et al., 2018; Wang, et al., 2006). The acid treatment has been used as methodology to obtain SiO_2_ with high purity (Baek et al., 2017), which has been used as starting material to the synthesis of polysiloxanes (Habaue et al., 2008), zeolites (Petkowicz et al., 2008; Saada et al., 2009) or ordered porous silicas (Schwanke et al., 2013).

**Table 2:** Chemical composition, expressed as an atomic percentage, of the starting chrysotile together with the materials obtained after acid treatment at 8, 12, 16 and 20 min

	Atomic %						
	Mg	Al	Si	Ca		Mg + Al	(Mg + Al)/Si
Natural	19.74	0.91	14.1	0.78		20.65	1.46
8 min	8.74	2.16	21.47	0.00		10.90	0.51
12 min	7.31	1.94	24.08	0.00		9.25	0.38
16 min	6.03	1.12	25.33	0.00		7.15	0.28
20 min	2.76	0.34	25.98	0.00		3.10	0.12

**Fig. 5:**
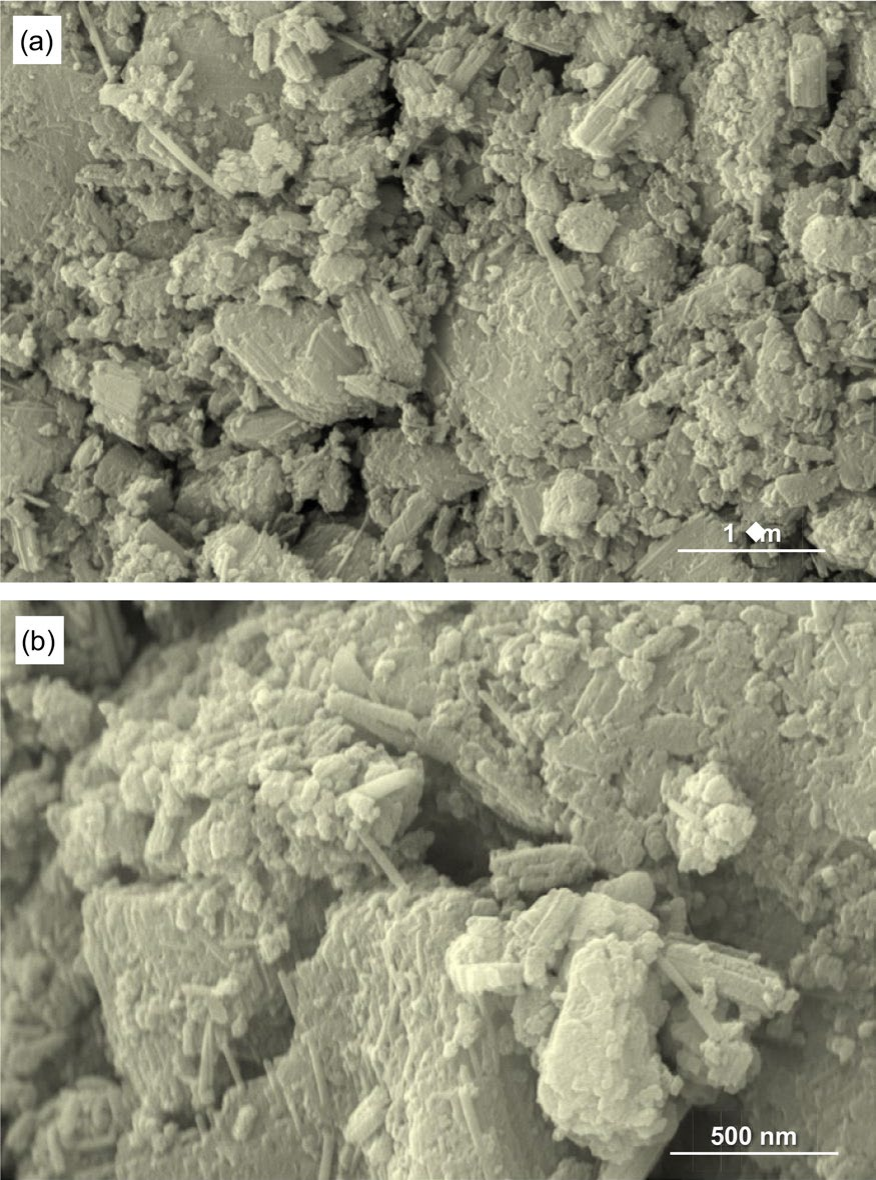
SEM images at different magnifications of 20 min-treated chrysotile fibers after the gentle grinding process. Magnification scale; 1 µm (**A**) and 500 nm (**B**)

In order to confirm the fragility of the treated samples, the chrysotile modified by microwave-assisted acid treatments during 20 min were subjected to an effortless grinding process in an agate mortar for 1 min. The morphology of the resulting sample was examined by SEM (Fig. 5). A completely different morphology compared to those for the natural chrysotile is clearly observed. Granulated particles with very jagged contours are distinguished, which are mostly associated creating porous aggregates. No vestiges of the initial fibrous morphology remain in the treated samples. Thus, the natural chrysotile fibers can be transformed in a porous and easily breakable scaffold after only 20 min of microwave-assisted acid treatment, as shown SEM images with different magnifications (Fig. 5).

Several studies have demonstrated the successful elimination of chrysotile asbestos through different treatment, resulting in the complete transformation of its fibers (Paolini et al., 2019). A representative example of the different types of procedures for the inactivation of asbestos and derivatives, as well as the treatment times, are presented in Table 3. All these approaches provide effective means of managing asbestos-containing materials, ensuring their safe disposal and reducing the risks associated with asbestos exposure. However, the processes employed require high temperatures (1400–1600 ºC) and/or considerable time (from several hours to several months), which hampers their marketability and industrially viability. It is worth noting that the microwave-assisted acid treatment proposed in this work is among the faster procedure reported in the literature for the inactivation of chrysotile fibers, presenting immense advantages in the waste management of asbestos, specially at industrial level.

**Table 3:** Procedure and treatment time for the inactivation of asbestos

Sample	Procedure	Time/Temp	Ref
Chrysotile fibers	Microwave-assisted acid treatment	8–20 min/100 ºC	This work
Chrysotile fibers and cement	Thermochemical treatment	10–24 h/100 ºC	Nam et al. (2014)
Asbestos-cement	High-energy milling	1–3 h/25 ºC	Iwaszko et al. (2018a)
Asbestos-cement waste	Vitrification process	90 min/1400 ºC	Iwaszko et al. (2018b)
Chrysotile asbestos	Acid leaching treatment	10 days/26 ºC	Valouma et al. (2017)
Chrysotile asbestos	Acid leaching treatment	9 days/25 ºC	Rozalen and Huertas (2013)
Chrysotile asbestos	Acid leaching treatment	16 h/25ºC	Sugama et al. (1998)
Cement-asbestos	Vitrification	5 h/1600 ºC	Dellisanti et al. (2009)
Amosite	Grinding/Thermal treatment	10 min/800 ºC	Bloise et al. (2018)
Crocidolite	Grinding/Thermal treatment	10 min/850 ºC	Bloise et al. (2018)
Chrysotile	Grinding/Thermal treatment	10 min/820 ºC	Bloise et al. (2018)
Chrysotile	Thermal treatment	830 ºC	Kusiorowski et al. (2012)
Asbestos-containing materials	Thermal treatment	1000 ºC	Kusiorowski et al. (2013)
Actinolite	Thermal treatment	1030 ºC	Bloise (2023b)
Tremolite	Thermal treatment	1000–1150 ºC	Bloise (2023a, 2023b)
Asbestos fibres	Thermal treatment	1100ºC	Bloise et al. (2016)
Cement-asbestos	Thermal treatment	1200ºC	Gualtieri and Boccaletti (2011)
Cement-asbestos	Thermal treatment	1200ºC	Gualtieri et al. (2011)
Cement-asbestos	Thermal treatment	750ºC	Gualtieri et al. (2012)
Chrysotile fibres	Biological technique	15 months	Favero-Longo et al. (2007)

## Conclusions

The present work proposes a novel procedure to modify the morphological and crystallo-chemical characteristics of chrysotile using microwave-assisted acid treatments. These treatments have eliminated octahedral layer cations of the chrysotile structure in only a few minutes, producing the destruction of the original 1:1 structure and the decrease of the flexibility of the fibers, while forming amorphous silica. The amorphous phase forms part of the remaining fibers, leaving a skeleton that has maintained an elongated morphology with very rounded edges and very porous surfaces. In fact, mesoporosity has varied from 10, in natural chrysotile, to 249 m^2^ g^−1^ after only 16 min of treatment. These skeletons of amorphous silica have not retained the physical resistance of the natural chrysotile fibers and are easily breakable, suggesting that the pathogenicity of the fibers has been inactivated. Although, a more in-depth study would be necessary, these results indicate that microwave-assisted acid treatments can be an economical, fast and effective alternative in the management and transformation of the hazardous asbestos waste.

## Supplementary Information

Below is the link to the electronic supplementary material.

Supplementary file1 (DOCX 2896 KB)

## References

[CR1] Amelo, B. V. (2012). X’Pert HighScore Plus Software, v3.0e, PANalytical. The Netherlands.

[CR2] Anbalagan, G., Sivakumar, G., Prabakaran, A. R., & Gunasekaran, S. (2010). Spectroscopic characterization of natural chrysotile. *Vibrational Spectroscopy,**52*, 122–127. 10.1016/j.vibspec.2009.11.007

[CR3] Baek, J., Jo, Y., Lee, J., Choi, S., Jeong, H., & Roh, Y. (2017). Synthesis of nano-sized Silicon oxide, iron oxide and carbonates from chrysotile asbestos. *Journal of Nanoscience Nanotechnology,**17*, 2610–2612. 10.1166/jnn.2017.13343 29664246 10.1166/jnn.2017.13343

[CR4] Bloise, A. (2023a). On the thermal breakdown of tremolite: A new method for distinguishing between asbestos and non-asbestos tremolite samples. *Journal of Materials Science,**58*, 8779–8795. 10.1007/s10853-023-08595-0

[CR5] Bloise, A. (2023b). Thermal behaviour of actinolite asbestos. *Journal of Materials Science,**54*, 11784–11795. 10.1007/s10853-019-03738-8

[CR6] Bloise, A., Catalano, M., Barrese, E., Gualtieri, A. F., Gandolfi, N. B., Capella, S., & Belluso, E. (2016). TG/DSC study of the thermal behaviour of hazardous mineral fibres. *Journal of Thermal Analysis and Calorimetry,**123*, 2225–2239. 10.1007/s10973-015-4939-8

[CR7] Bloise, A., Catalano, M., & Gualtieri, A. F. (2018). Effect of grinding on chrysotile, amosite and crocidolite and implications for thermal treatment. *Minerals,**8*, 135. 10.3390/min8040135

[CR8] Brown, G. T., & Brindley, G. W. (1980). *X-Ray Diffraction Procedures for Clay Mineral Identification*. 10.1180/mono-5.5

[CR9] Brunauer, S., Emmett, P. H., & Teller, E. (1938). Adsorption of gases in multimolecular layers. *Journal of American Chemical Society,**60*, 309–319. 10.1021/ja01269a023

[CR10] Cortés, J. R. C. (2003). Cáncer y Apoptosis. Vertientes. *Revista Especializada En Ciencias De La Salud,**6*, 8–14.

[CR11] Cozak, D., Barbeau, C., Gauvin, F., Barry, J. P., DeBlois, C., & DeWolf, R. (1983). The reaction of chrysotile asbestos with titanium(III) chloride. Characterization of the reaction products. *Canadian Journal of Chemistry*, *61*, 2753. 10.1139/v83-473.

[CR12] Dellisanti, F., Rossi, P. L., & Valdrè, G. (2009). Remediation of asbestos containing materials by Joule heating vitrification performed in a pre-pilot apparatus. *International Journal of Mineral Processing,**91*, 61–67. 10.1016/j.minpro.2008.12.001

[CR13] Dogan, A. U., Dogan, M., Onal, M., Sarikaya, Y., Aburub, A., & Wurster, D. E. (2006). Baseline studies of the clay minerals society source clays: Specific surface area by the Brunauer Emmett Teller (BET) method. *Clays and Clay Minerals,**54*, 62–66. 10.1346/CCMM.2006.0540108

[CR14] Douglas, T., & Van den Borre, L. (2019). Asbestos neglect: Why asbestos exposure deserves greater policy attention. *Health Policy,**123*, 516–519. 10.1016/j.healthpol.2019.02.001 30770142 10.1016/j.healthpol.2019.02.001

[CR15] Favero-Longo, S. E., Girlanda, M., Honegger, R., Fubini, B., & Piervittori, R. (2007). Interactions of sterile-cultured lichen-forming ascomycetes with asbestos fibers. *Mycological Research,**111*, 473–481. 10.1016/j.healthpol.2019.02.001 17512715 10.1016/j.mycres.2007.01.013

[CR16] Fernández, L. C., Alvarez, R. F., González-Barcala, F. J., & Portal, J. A. R. (2013). Contaminación del aire interior y su impacto en la patología respiratoria. *Archivos De Bronconeumología,**49*, 22–27. 10.1016/j.arbres.2012.04.005 22704531 10.1016/j.arbres.2012.04.005

[CR17] Franco, F., Cecilia, J. A., Pozo, M., Pardo, L., Bellido, E., & García-Sancho, C. (2020). Microwave assisted acid treatment of kerolitic clays from the Neogene Madrid Basin (Spain) and its use in CO_2_ capture processes. *Microporous and Mesoporous Materials,**292*, 109749. 10.1016/j.micromeso.2019.109749

[CR18] Franco, F., Pérez-Maqueda, L. A., Ramírez-Valle, V., & Pérez-Rodríguez, J. L. (2006). Spectroscopic study of the dehydroxylation process of a sonicated antigorite. *European Journal of Mineralogy,**18*, 257–264. 10.1127/0935-1221/2006/0018-0257

[CR19] Franco, F., Pozo, M., Cecilia, J. A., Benítez-Guerrero, M., & Lorente, M. (2016). Effectiveness of microwave assisted acid treatment on dioctahedral and trioctahedral smectites. The influence of octahedral composition. *Applied Clay Science,**120*, 70–80. 10.1016/j.clay.2015.11.021

[CR20] Franco, F., Pozo, M., Cecilia, J. A., Benítez-Guerrero, M., Pozo, E., & Martín Rubí, J. A. (2014). Microwave assisted acid treatment of sepiolite: The role of composition and “crystallinity.” *Applied Clay Science,**102*, 15–27. 10.1016/j.clay.2014.10.013

[CR21] Goni, J., Thomasssin, J. H., Jaurand, M. C., & Touray, J. C. (1979). Photoelectron spectroscopy analysis of asbestos dissolution in acidic media of biological interest. *Physics and Chemistry of the Earth,**11*, 807–817. 10.1016/0079-1946(79)90075-2

[CR22] Granat, K., Nowak, D., Pigiel, M., Florczak, W., & Opyd, B. (2015). Application of microwave radiation in innovative process of neutralising asbestos-containing wastes. *Archives of Civil and Mechanical Engineering,**15*, 188–194. 10.1016/j.acme.2014.05.012

[CR23] Gualtieri, A. F., & Boccaletti, M. (2011). Recycling of the product of thermal inertization of cement–asbestos for the production of concrete. *Construction and Building Materials,**25*, 3561–3569. 10.1016/j.conbuildmat.2011.03.049

[CR24] Gualtieri, A. F., Giacobbe, C., Sardisco, L., Saraceno, M., Gualtieri, M. L., Lusvardi, G., Cavenati, C., & Zanatto, I. (2011). Recycling of the product of thermal inertization of cement–asbestos for various industrial applications. *Waste Management,**31*, 91–100. 10.1016/j.wasman.2010.07.006 20708915 10.1016/j.wasman.2010.07.006

[CR25] Gualtieri, A. F., Veratti, L., Tucci, A., & Esposito, L. (2012). Recycling of the product of thermal inertization of cement-asbestos in geopolymers. *Construction and Building Materials,**31*, 47–51. 10.1016/j.conbuildmat.2011.12.087

[CR26] Habaue, S., Sato, K., Yamashita, K., Shimamura, T., Kaito, M., Masuda, T., & Kajiwara, M. (2008). Polysiloxanes derived from chrysotile asbestos via acid-leaching and silylation processes. *Journal of Applied Polymer Science,**110*, 2891–2897. 10.1002/app.28899

[CR27] Harington, J. S., Allison, A. C., & Badami, D. V. (1975). Mineral fibers: Chemical, physicochemical, and biological properties. *Advances in Pharmacology,**12*, 291–402. 10.1016/S1054-3589(08)60223-9 10.1016/s1054-3589(08)60223-9 1098431

[CR28] Harington, J. S., Miller, K., & Macnab, G. (1971). Hemolysis by asbestos. *Environmental Research,**4*, 95–117. 10.1016/0013-9351(71)90038-7 4997300 10.1016/0013-9351(71)90038-7

[CR29] Health, C. (1983). Asbestos: The blue, the brown, and the white. *Lancet,**322*, 349. 10.1016/S0140-6736(83)90332-X

[CR30] Hongo, T. (2016). Dissolution of the chrysotile structure in nitric-acid solutions at different pH. *Clay Minerals,**51*, 715–722. 10.1180/claymin.2016.051.5.02

[CR31] Iwaszko, J., Zawada, A., & Lubas, M. (2018a). Influence of high-energy milling on structure and microstructure of asbestos-cement materials. *Journal of Molecular Structure,**1155*, 51–57. 10.1016/j.molstruc.2017.10.104

[CR32] Iwaszko, J., Zawada, A., Przerada, I., & Lubas, M. (2018b). Structural and microstructural aspects of asbestos-cement waste vitrification. *Spectrochimica Acta Part a: Molecular and Biomolecular Spectroscopy,**195*, 95–102. 10.1016/j.saa.2018.01.053 29414587 10.1016/j.saa.2018.01.053

[CR33] Jaurand, M. C., Magne, L., Boulner, J. L., & Bignon, J. (1981). In vitro reactivity of alveolar macrophages and red blood cells with asbestos fibres treated with oxalic acid, sulfur dioxide and benzo-3,4-pyrene. *Toxicology,**21*, 323–342. 10.1016/0300-483X(81)90147-5 6272445 10.1016/0300-483x(81)90147-5

[CR34] Komadel, P. (2016). Acid activated clays: Materials in continuous demand. *Applied Clay Science,**131*, 84–99. 10.1016/j.clay.2016.05.001

[CR35] Korichi, S., Elias, A., & Mefti, A. (2009). Characterization of smectite after acid activation with microwave irradiation. *Applied Clay Science,**42*, 432–438. 10.1016/j.clay.2008.04.014

[CR36] Kusiorowski, R., Zaremba, T., Piotrowski, J., & Adamek, J. (2012). Thermal decomposition of different types of asbestos. *Journal of Thermal Analysis and Calorimetry,**109*, 693–704. 10.1007/s10973-012-2222-9

[CR37] Kusiorowski, R., Zaremba, T., Piotrowski, J., & Gerle, A. (2013). Thermal decomposition of asbestos-containing materials. *Journal of Thermal Analysis and Calorimetry,**113*, 179–188. 10.1007/s10973-013-3038-y

[CR38] Landrigan, P. J., Fuller, R., Fisher, S., Suk, W. A., Sly, P., Chiles, T. C., & Bose-O’Reilly, S. (2019). Pollution and children’s health. *Science of the Total Environment,**650*, 2389–2394. 10.1016/j.scitotenv.2018.09.375 30292994 10.1016/j.scitotenv.2018.09.375

[CR39] Langer, A., Nolan, R., & Addison, J. (1991). Physico-chemical properties of asbestos as determinants of biological potential. *Chap,**17*, 211–229.

[CR40] Larson, A. C., & Dreele, R. B. V. (1994). General structure analysis system (GSAS) Software, Los Alamos National Lab, Rep. No. LA-UR-86748.

[CR41] Lemen, R. A. (2004). Asbestos in brakes: Exposure and risk of disease. *American Journal of Industrial Medicine,**45*, 229–237. 10.1002/ajim.10334 14991849 10.1002/ajim.10334

[CR42] Maletaskic, J., Stankovic, N., Daneu, N., Babic, B., Stoiljkovic, M., Yoshida, K., & Matovic, B. (2018). Acid leaching of natural chrysotile asbestos to mesoporous silica fibers. *Physics and Chemistry of Minerals,**45*, 343–351. 10.1007/s00269-017-0924-z

[CR43] Mazzeo, A. (2018). The temporalities of asbestos mining and community activism. *The Extractive Industries and Society,**5*, 223–229. 10.1016/j.exis.2018.02.004

[CR44] Nam, S. N., Jeong, S., & Lim, H. (2014). Thermochemical destruction of asbestos-containing roofing slate and the feasibility of using recycled waste sulfuric acid. *Journal of Hazardous Materials,**265*, 151–157. 10.1016/j.jhazmat.2013.11.004 24361492 10.1016/j.jhazmat.2013.11.004

[CR45] Naumova, L. N., Pavlenko, V. I., & Cherkashina, N. I. (2019). Modification of chrysotile fiber surface and its effect on the physical and mechanical characteristics of chrysotile cement. *Protection of Metals and Physical Chemistry of Surfaces,**55*, 330–334. 10.1134/S2070205119020229

[CR46] Osthaus, B. (1953). Chemical determination of tetrahedral ions in nontronite and montmorillonite. *Clays and Clay Minerals,**2*, 404–417. 10.1346/CCMN.1953.0020134

[CR47] Osthaus, B. (1955). Kinetic studies on montmorillonites and nontronite by the acid-dissolution technique. *Clays and Clay Minerals,**4*, 301–321. 10.1346/CCMN.1955.0040134

[CR48] Paolini, V., Tomassetti, L., Segreto, M., Borin, D., Liotta, F., Torre, M., & Petracchini, F. (2019). Asbestos treatment technologies. *Journal of Material Cycles and Waste Management,**21*, 205–226. 10.1007/s10163-018-0793-7

[CR49] Pardo-Canales, L., Essih, S., Cecilia, J.A., Domínguez-Maqueda, M., Olmo-Sánchez, M.I., Pozo-Rodríguez, M., Franco, F. (2020). Modification of the textural properties of palygorskite through microwave assisted acid treatment. Influence of the octahedral sheet composition. *Applied Clay Science*, *196*, 105745. 10.1016/j.clay.2020.105745.

[CR50] Park, S. H. (2018). Types and health hazards of fibrous materials used as asbestos substitutes. *Safety and Health at Work,**9*, 360–364. 10.1016/j.shaw.2018.05.001 30370171 10.1016/j.shaw.2018.05.001 PMC6129992

[CR51] Pawelczyk, A., Bozek, F., Grabas, K., & Checmanowski, J. (2017). Chemical elimination of the harmful properties of asbestos from military facilities. *Waste Management,**61*, 377–385. 10.1016/j.wasman.2016.11.041 27979425 10.1016/j.wasman.2016.11.041

[CR52] Peña-Castro, M., Montero-Acosta, M., & Saba, M. (2023). A critical review of asbestos concentrations in water and air, according to exposure sources. *Heliyon,**9*, e15730. 10.1016/j.heliyon.2023.e15730 37305461 10.1016/j.heliyon.2023.e15730 PMC10256854

[CR53] Petkowicz, D. I., Rigo, R. T., Radtke, C., dos Pergher, S. B., & Santos, J. H. Z. (2008). Zeolite NaA from Brazilian chrysotile and rice husk. *Microporous and Mesoporous Materials,**116*, 548–544. 10.1016/j.micromeso.2008.05.014

[CR54] Rozalen, M., & Huertas, F. J. (2013). Comparative effect of chrysotile leaching in nitric, sulfuric and oxalic acids at room temperature. *Chemical Geology,**352*, 134–142. 10.1016/j.chemgeo.2013.06.004

[CR55] Rudd, R. (2008). Asbestos and the lung. *Medicine,**36*, 261–264. 10.1016/j.mpmed.2008.02.001

[CR56] Ruíz Cruz, M. D., & Franco, F. (2000). Thermal behavior of the kaolinite-hydrazine intercalation complex. *Clays and Clay Minerals,**48*, 63–67. 10.1346/CCMN.2000.0480108

[CR57] Saada, M. A., Soulard, M., Patarin, J., & Regis, R. C. (2009). Synthesis of zeolite materials from asbestos wastes: An economical approach. *Microporous and Mesoporous Materials,**122*, 275–282. 10.1016/j.micromeso.2009.03.011

[CR58] Schwanke, A. J., Lopes, C. W., & Pergher, S. B. C. (2013). Synthesis of mesoporous material from chrysotile-derived silica. *Materials Sciences and Applications,**4*, 68–72. 10.4236/msa.2013.48A009

[CR59] Sing, K. S. W., Everett, D. H., Haul, R. A. W., Moscou, L., Pieroti, R. A., Rouquerol, J., & Siemieniewska, T. (1985). Reporting physisorption data for gas/solid systems with special reference to the determination of surface area and porosity (Recommendations 1984). *Pure and Applied Chemistry,**57*, 603–619. 10.1351/pac198557040603

[CR60] Spasiano, D., & Pirozzi, F. (2017). Treatments of asbestos containing wastes. *Journal of Environmental Management,**204*, 82–91. 10.1016/j.jenvman.2017.08.038 28863339 10.1016/j.jenvman.2017.08.038

[CR61] Sugama, T., Sabatini, R., & Petrakis, L. (1998). Decomposition of chrysotile asbestos by fluorosulfonic acid. *Industrial & Engineering Chemistry Research,**37*, 79–88. 10.1021/ie9702744

[CR62] Suzuki, Y., Yuen, S. R., & Ashley, R. (2005). Short, thin asbestos fibers contribute to the development of human malignant mesothelioma: Pathological evidence. *International Journal of Hygiene and Environmental Health,**208*, 201–210. 10.1016/j.ijheh.2005.01.015 15971859 10.1016/j.ijheh.2005.01.015

[CR63] Thives, L. P., Ghisi, E., Thives, J. J., & Silva Vieira, A. (2022). Is asbestos still a problem in the world? A current review. *Journal of Environmental Management,**319*, 115716. 10.1016/j.jenvman.2022.115716 35863303 10.1016/j.jenvman.2022.115716

[CR64] Thommes, M., Kaneko, K., Neimark, A., Olivier, J., Rodriguez-Reinoso, F., Rouquerol, J., & Sing, K. (2015). Physisorption of gases, with special reference to the evaluation of surface area and pore size distribution (IUPAC Technical Report). *Pure and Applied Chemistry,**87*, 1051–1069. 10.1515/pac-2014-1117

[CR65] Valouma, A., Verganelaki, A., Tetoros, I., Maravelaki-Kalaitzaki, P., & Gidarakos, E. (2017). Magnesium oxide production from chrysotile asbestos detoxification with oxalic acid treatment. *Journal of Hazardous Materials,**336*, 93–100. 10.1016/j.jhazmat.2017.04.019 28477559 10.1016/j.jhazmat.2017.04.019

[CR66] Wang, L., Lu, A., Wang, C., Zheng, X., Zhao, D., & Liu, R. (2006). Nano-fibriform production of silica from natural chrysotile. *Journal of Colloid and Interface Science,**295*, 436–439. 10.1016/j.jcis.2005.08.055 16212972 10.1016/j.jcis.2005.08.055

[CR67] Yarborough, C. M. (2007). The risk of mesothelioma from exposure to chrysotile asbestos. *Current Opinion in Pulmonary Medicine,**13*, 334–338. 10.1097/MCP.0b013e328121446c 17534182 10.1097/MCP.0b013e328121446c

[CR68] Yoshikawa, N., Kashimura, K., Hashiguchi, M., Sato, M., Horikoshi, S., Mitani, T., & Shinohara, N. (2015). Detoxification mechanism of asbestos materials by microwave treatment. *Journal of Hazardous Materials,**284*, 201–206. 10.1016/j.jhazmat.2014.09.030 25463234 10.1016/j.jhazmat.2014.09.030

[CR69] Zaghloul, H. H., & Circeo, L. J. (1993). Destruction and vitrification of asbestos using plasma arc technology. USACERL Technical Report CPAR-TR-EP-93A10.

