# Supplementary material for: Microwave-assisted acid treatment for the mineral transformation of chrysotile as an alternative for asbestos waste management
Source: Environ Geochem Health. 2024 Jul 18;46(9):332. doi: 10.1007/s10653-024-01993-6 (PMC11258100; doi:10.1007/s10653-024-01993-6)
Supplement: Supplementary file 1 — Supplementary file1 (DOCX 2896 KB) [file 10653_2024_1993_MOESM1_ESM.docx]

**Microwave-assisted acid treatment for the mineral transformation of** **chrysotile as an alternative for asbestos waste management**

**Supplementary material**

**Figure S1.** (a) Natural chrysotile before the treatment. (b) Chrysotile in nitric acid after microwave treatment.


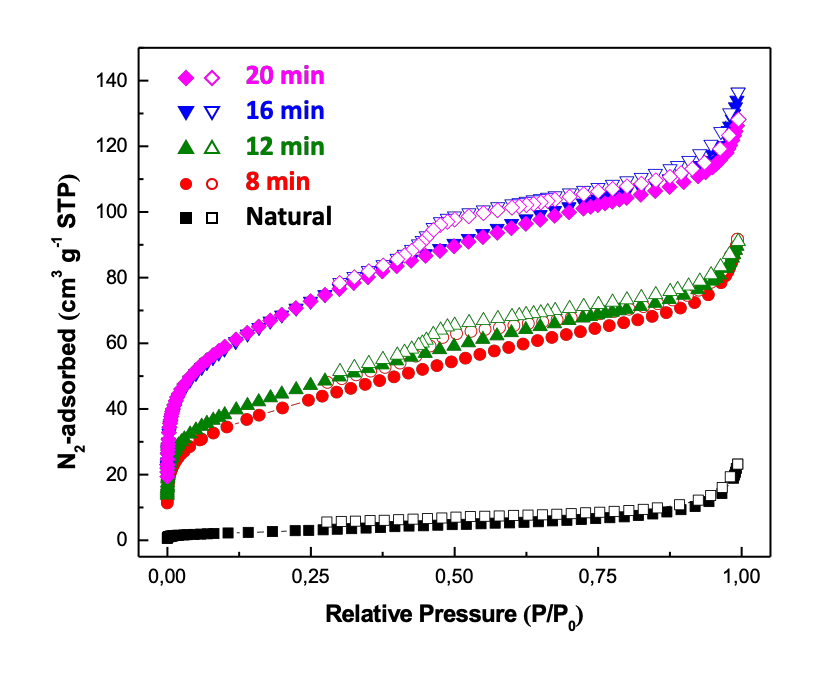


**Figure S2.** Isotherms of N_2_ adsorption on chrysotile fibers and materials obtained after microwave-assisted acid treatments.
